# High throughput nanostructure-initiator mass spectrometry screening of microbial growth conditions for maximal β-glucosidase production

**DOI:** 10.3389/fmicb.2013.00365

**Published:** 2013-12-06

**Authors:** Xiaoliang Cheng, Jennifer Hiras, Kai Deng, Benjamin Bowen, Blake A. Simmons, Paul D. Adams, Steven W. Singer, Trent R. Northen

**Affiliations:** ^1^Technology Division, Joint BioEnergy InstituteEmeryville, CA, USA; ^2^Department of Bioenergy/GTL & Structural Biology, Life Sciences Division, Lawrence Berkeley National LaboratoryBerkeley, CA, USA; ^3^Deconstruction Division, Joint BioEnergy InstituteEmeryville, CA, USA; ^4^Physical Biosciences Division, Lawrence Berkeley National LaboratoryBerkeley, CA, USA; ^5^Biological and Materials Science Center, Sandia National LaboratoriesLivermore, CA, USA; ^6^Department of Geochemistry, Earth Sciences Division, Lawrence Berkeley National LaboratoryBerkeley, CA, USA; ^7^Department of Ecology, Earth Sciences Division, Lawrence Berkeley National LaboratoryBerkeley, CA, USA

**Keywords:** NIMS, high throughput, β-glucosidase, enzymatic activity screening, microbial communities

## Abstract

Production of biofuels via enzymatic hydrolysis of complex plant polysaccharides is a subject of intense global interest. Microbial communities are known to express a wide range of enzymes necessary for the saccharification of lignocellulosic feedstocks and serve as a powerful reservoir for enzyme discovery. However, the growth temperature and conditions that yield high cellulase activity vary widely, and the throughput to identify optimal conditions has been limited by the slow handling and conventional analysis. A rapid method that uses small volumes of isolate culture to resolve specific enzyme activity is needed. In this work, a high throughput nanostructure-initiator mass spectrometry (NIMS)-based approach was developed for screening a thermophilic cellulolytic actinomycete, *Thermobispora bispora*, for β-glucosidase production under various growth conditions. Media that produced high β-glucosidase activity were found to be I/S + glucose or microcrystalline cellulose (MCC), Medium 84 + rolled oats, and M9TE + MCC at 45°C. Supernatants of cell cultures grown in M9TE + 1% MCC cleaved 2.5 times more substrate at 45°C than at all other temperatures. While *T. bispora* is reported to grow optimally at 60°C in Medium 84 + rolled oats and M9TE + 1% MCC, approximately 40% more conversion was observed at 45°C. This high throughput NIMS approach may provide an important tool in discovery and characterization of enzymes from environmental microbes for industrial and biofuel applications.

## INTRODUCTION

Saccharification of lignocellulosic feedstocks has great potential to provide fermentable sugars for production of renewable and potentially carbon neutral biofuels. Significant efforts are underway to determine cost-effective processes to enzymatically hydrolyze these complex and often recalcitrant materials (Helenius and Aebi, 2001; Lynd et al., 2002; Shallom and Shoham, 2003; Doi and Kosugi, 2004; Blanch et al., 2008; Dashtban et al., 2009; Pauly and Keegstra, 2010; Steen et al., 2010). However, a large number of existing industrial enzymes are not compatible with many highly effective pre-treatment strategies, such as high temperatures and ionic liquid pre-treatment, and require substantial post-processing in order to make these substrates amenable to their use. Therefore, there is an urgent need for the discovery and development of high performance enzymes under these pre-treatment conditions in order to minimize costly post-processing steps (Blanch et al., 2008; Steen et al., 2010).

Bacterial glycoside hydrolase (GH) enzymes perform under a wide spectrum of conditions (Lynd et al., 2002). Aerobic cellulolytic actinobacteria have been shown to degrade cellulose via combinations of soluble cellulases and hemicellulases (Lynd et al., 2002). *Thermobispora* (formerly *Microbispora*) *bispora* R51 (DSM 43833) is thermophilic, Gram positive actinobacterium known to degrade cellulose with high levels of efficiency and a complete cellulase complex has been identified in the genome of the *T. bispora*type strain (DSM 43833; Waldron et al., 1986; Liolios et al., 2010). However, ideal growth conditions for *T. bispora*have not yet been elucidated. Reports of optimal growth temperature (45–60°C) and conditions that yield high cellulase activity vary widely (Liolios et al., 2010; Anderson et al., 2012). Methods are therefore needed to determine optimal growth conditions for *T. bispora*that result in sufficient enzyme expression for activity analysis. Ideally, these methods would be suitable for use of small volumes of isolate culture, could be performed on crude microbial cultures and would be suitable for resolving specific enzyme activities, most of which are based on spectroscopic properties changes of a substrate upon hydrolysis (Sharrock, 1988; Coleman et al., 2007, 2010; Dashtban et al., 2010). Recently, self-assembled monolayers for matrix assisted laser desorption/ionization time-of-flight (SAMDI-TOF) mass spectrometry was used for the analysis of β-1,4-galactosidase activities on gold surfaces (Su and Mrksich, 2002; Ban and Mrksich, 2008 and fluorous-phase-chemistry were used to study GHs on aluminum oxide-coated glass slides (Chang et al., 2010). However, the reaction conditions for surface-based techniques are often complex to handle and hard to control, especially at high temperatures. Nanostructure-initiator mass spectrometry (NIMS; Northen et al., 2007, 2008; Reindl and Northen, 2010; Reindl et al., 2011; de Rond et al., 2013) has shown to be suitable for analysis of crude microbial communities. We have recently found that automation of the liquid handling using acoustic printing to transfer sample from multiwell microtiter plates onto the NIMS chips results in comparable performance but in 100 times higher throughput than traditional manual spotting (Greving et al., 2012). Compared to other mass spectrometry-based methods, this method consumes far less solvent. This technique may be applied to rapidly determine optimal temperature and culture medium for *T. bispora*GH production.

Here, we describe a method of screening *T. bispora* for β-glucosidase, a key GH enzyme found in the cellulase mixtures that hydrolyzes cellobiose to glucose (Lynd et al., 2002), under various growth conditions by using acoustic printing coupled to NIMS. *T. bispora* was grown in 24-well plates at various temperatures, times, and with different cellulosic biomass as carbon sources [microcrystalline cellulose (MCC), ammonium fiber expansion (AFEX)-pretreated switchgrass, rolled oats, or glucose]. Acoustic NIMS analysis was performed on all culture conditions to evaluate conditions resulting in highest enzyme activities.

## METHODS

### NIMS SUBSTRATE PREPARATION

The NIMS substrate used in this study was cellobiose attached to a perfluorinated tag (Reindl et al., 2011; Deng et al., 2012). Cellobiose was purchased from Sigma-Aldrich (St. Louis, MO, USA). Substrate synthesis is described elsewhere (Reindl et al., 2011; Deng et al., 2012). Briefly, the (CH_2_)_5_-linker was coupled to the reducing end of the oligosaccharides using Schmidt imidate chemistry. Hydrogenation using Pd/C was used to remove the carbobenzyloxy (Cbz) protection group to give a primary amine. Subsequently the heptadecafluoro-1,1,2,2-tetrahydrodecyl (F17) tag was attached to a dimethyl-arginine using an amide bond forming reaction. Finally, peptide coupling is used to link the sugar moiety with the fluorous tag to yield the desired substrate.

### FABRICATION OF NIMS CHIP

The production of NIMS chips has been described in great detail elsewhere (Northen et al., 2008; Woo et al., 2008). In brief, single-sided polished P/Boron, orientation <1-0-0>, resistivity 0.01–0.02 Ω cm, thickness 525 ± 25 μm 4″ silicon wafers were obtained from Silicon Quest International (Santa Clara, CA, USA). A 70 mm × 70 mm square was cut from this wafer and cleaned thoroughly with methanol, followed by anodic etching with 25% hydrofluoric acid in LC-MS grade ethanol (Fisher Scientific, Waltham, MA, USA) in a custom Teflon etching chamber [EXTREME CAUTION IS REQUIRED]. Throughout the etching process, 2.3 A was applied for 15 min. After etching, the chips were coated by adding 250 μL of the initiator liquid bis(heptadecafluoro-1,1,2,2-tetrahydrodecyl)tetramethyl-disiloxane (Gelest Morrisville, PA, USA) for 20 min and the excess initiator was blown off with a jet of nitrogen.

### CELL CULTURE

*T. bispora* R51 (DSM 43833) was purchased from the German Collection of Microorganisms and Cell Cultures (DSMZ, ). Liquid cultures were grown in DSMZ Medium 65 at 45°C overnight while shaking at 250 rpm (DSMZ direct correspondence). Cell density was measured using a SpectraMax M2 spectrophotometer (Molecular Devices, Sunnyvale, CA, USA). At OD_600_ = 0.500, cells were washed three times to remove excess glucose from culture medium. The starter culture was transferred to a sterile 15 mL conical tube and centrifuged for 5 min at 4000 rpm, then resuspended in glucose-free Medium 65. Experimental cultures were established in 24-well, round well bottom plates (Whatman, Maidstone, UK) and sealed with BugStopper^TM^ microplate capmats (Whatman, Maidstone, UK) to prevent evaporation. Duplicate wells per plate contained 5 mL Medium 65, I/S, M9TE, or Medium 84. Each culture contained 10 mM glucose, 1% AFEX-pretreated switchgrass or MCC (Sigma-Aldrich, St. Louis, MO, USA), or 2% rolled oats (Quaker, Chicago, IL, USA) as the carbon/energy source (**Table 1**). Wells were inoculated with 50 μL washed starter culture and incubated at 45, 50, 55, and 60°C while shaking at 850 rpm in a plate incubator. Media lacking cells or lacking both cells and a supplemental carbon/energy source were included as negative controls on each plate.

**Table 1:** *Thermobispora bispora* growth conditions tested in this study.

Medium	Carbon source	Rationale
DSMZ 65	Glucose Switchgrass MCC	DSMZ recommended *T. bispora* growth medium prepared with glucose
I/S	Glucose Switchgrass MCC	Previously reported actinobacteria isolation/screening medium prepared with MCC (Waldron and Eveleigh, 1986)
M9TE	MCC	Minimal medium + cellulose (Gladden et al., 2011)
DSMZ 84	Rolled oats	Previous DSMZ recommended *T. bispora* growth medium prepared with soluble polysaccharides from rolled oats

### ENZYMATIC ASSAYS

Samples (45 μL) were transferred to 384-well plates prefilled with 5 μL 200 μM NIMS probe per well at 3 and 24 h. The enzymatic reaction was incubated at 50°C for 1 h, and then quenched with cold methanol (50 μL). After 5 min, samples were centrifuged at 3000 rpm for 3 min, then 8 μL supernatant were transferred to 384-well acoustic plates (Greiner Bio-one, Germany) for printing.

### ACOUSTIC PRINTING

The assay mixture was acoustically printed onto a NIMS chip using EDC ATS-100 acoustic transfer system (EDC Biosystems, Fremont, CA, USA) with a sample deposition volume of 1 nL. Samples were printed with the microarray spot pitch (center-to-center distance) set at 450 μm. This format allowed ~2 samples/mm^2^ and the total assay deposition time for 288 samples generated in this work was less than 2 min.

### NIMS ANALYSIS

Nanostructure-initiator mass spectrometry was performed using an ABI/Sciex 5800 MALDI TOFTOF mass spectrum with laser intensity of 2500 over a mass range of 700–1500 Da. The data collection was controlled using MALDI MSI 4800 imaging tool, and each position on NIMS chip accumulated 18 laser shots and scanning step size was set at 75 μm step both vertically and horizontally. The total array acquisition time was 2 h. The enzymatic activity was measured based on the fractional conversion of the probe for each reaction. Briefly, signal intensities for probe and product from the acquired spectrum, activity was determined by calculating glucose formation as product/(probe + product) for each pixel over the image using an analysis algorithm written in Matlab and plotted as a false color image. Negative control of non-enzymatic hydrolysis was subtracted to correct the calculated activities, this approach minimize the effects of intensity across the surface. Then, a heatmap showing activities across all conditions was plotted.

## RESULTS AND DISCUSSION

**FIGURE 1:**
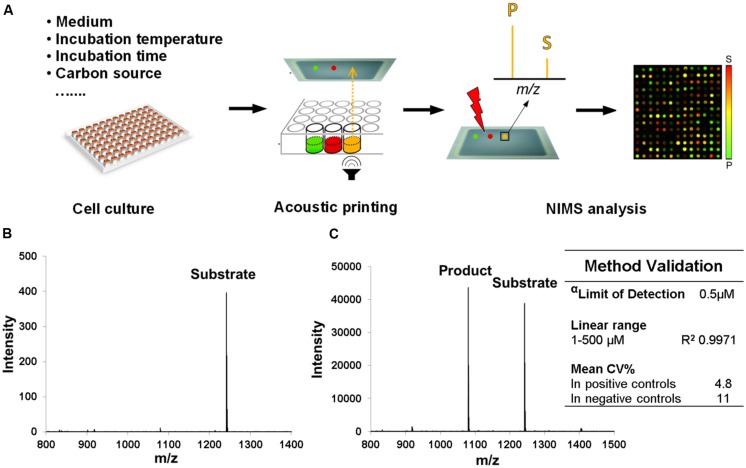
**Schematic of high throughput NIMS for screening growth conditions and method validation.** **(A)** Samples generated from various growth conditions were acoustically printed and analyzed by NIMS approach. **(B)** Spectrum of 1 μM substrate (0.5 μL sample was manually deposited, 500 fmoles), which is the limit of quantification (LOQ) in this method (S/N ratio >10). **(C)** Spectrum of an assay mixture that produced 55% conversion. The lowest concentration of substrate (α) is detected with S/N ratio >5 in this assay. The low coefficient of variance (CV) we found to be <20%.

A method of screening *T. bispora*, a known cellulose degrader, for β-glucosidase production under various growth conditions using acoustic printing coupled to NIMS was developed. Here, we tested the ability for *T. bispora* to grow on media containing a variety of energy sources and at specific temperatures (**Table 1**). Recipes were prepared following standard protocols or modified to include alternate energy sources (**Table 1**). Enzymatic assays were performed on all cultures using identical conditions and acoustic NIMS analysis was performed to evaluate conditions resulting in highest enzyme activities (**Figure 1**). The detection limit of the substrate is 0.5 μM in the assay mixture, which is more sensitive than conventional measurement of cellobiose and glucose (Reindl et al., 2011). A coefficient of variance (CV) <5% in positive controls assured the method was validated. The supernatants were screened for cleavage of a labeled cellobiose substrate to labeled glucose product.

**FIGURE 2:**
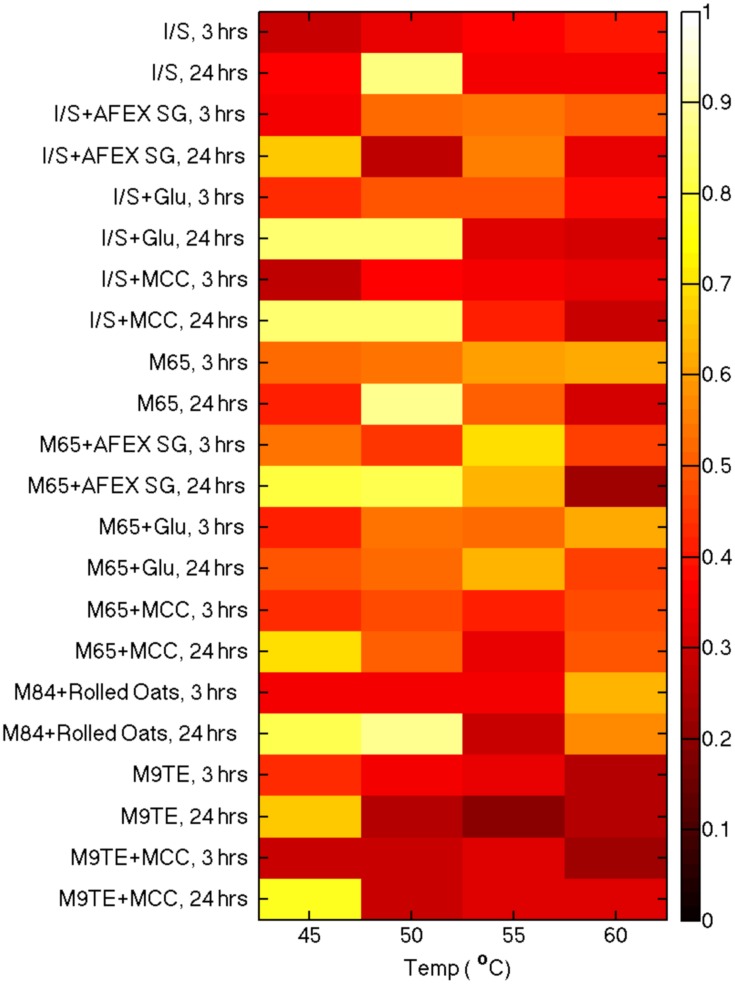
**Analysis of β-glucosidase production from cell culture supernatants grown in various media and at different temperatures.** Enzyme activities for the various growth conditions were compared using a cellobiose probe incubated at 50°C for 1 h.

Heatmaps depicting all conditions assayed for β-glucosidase activity are shown in **Figure 2**. Initial substrate conversion was observed to be minimal at the 3 h time point, demonstrating low production of β-glucosidase at an early culture time. We expected to detect changes in β-glucosidase activities after giving cell cultures an opportunity to grow overnight. After 24 h, the majority of all cultures grown at 45°C had higher conversion of substrate to product than their counterparts at 50,55, and 60°C, which suggests 45°C is the best incubation temperature for cellulase production.

**FIGURE 3:**
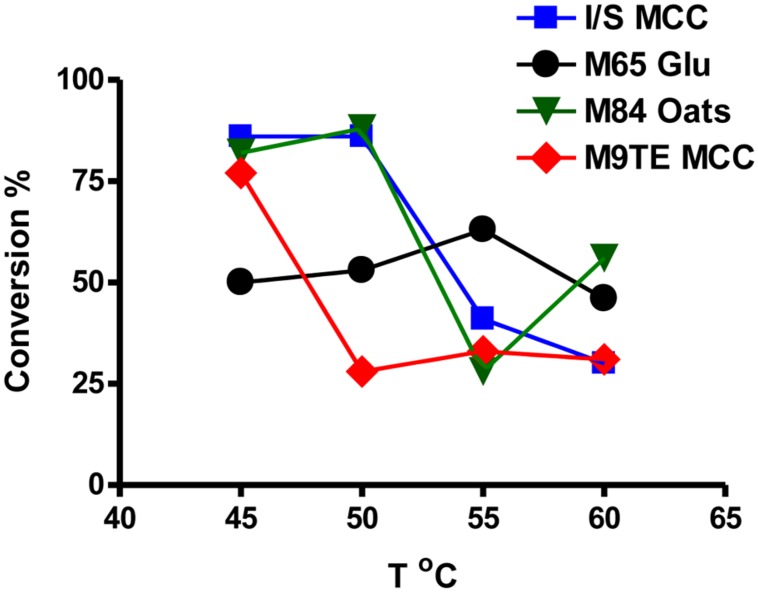
**Direct comparison of supernatants from cell cultures grown in media containing the recommended energy source after 24 h.** Squares, I/S + 1% MCC; circles, Medium 65 + 10 mM glucose; triangles, Medium 84 + rolled oats; diamonds, M9TE + 1% MCC.

Comparison of supernatants from cells grown on various reported media enabled the identification of optimal conditions for β-glucosidase production. Actinobacterial Isolation and Screening medium (I/S) and M9TE are typically prepared with 1% MCC as the cellulose substrate, DSMZ Medium 65 contains glucose, and DSMZ Medium 84 utilizes soluble β-glucans from boiling rolled oats. The reported growth temperature for bacteria on I/S, M9TE, and Medium 84 is 60°C, while growth on Medium 65 is stated as 45°C. However, this study found that cell culture supernatants grown in I/S (**Figure 3**, squares), Medium 84 (**Figure 3**, triangles), and M9TE (**Figure 3**, diamonds) had the highest conversions at 45°C. Supernatants of cell culture grown in Medium 65 (**Figure 3**, circles) converted between 50 and 60% of the substrate at all four growth temperatures. Supernatants of cell cultures grown in M9TE + 1% MCC cleaved 2.5 times more substrate at 45°C than at all other temperatures (**Figure 3**). At 60°C, growth in Medium 84 + rolled oats had the most conversion at this temperature, but not as much as 45 and 50°C cultures (**Figure 3**). While *T. bispora* is reported to grow optimally at 60°C in Medium 84 + rolled oats and M9TE + 1% MCC, approximately 40% more conversion was observed at 45°C (**Figure 3**). An interesting finding was that glucose appears to stimulate β-glucosidase production (**Figure 2**). It is a well-documented fact that glucose inhibits cellulases during enzymatic hydrolysis (Xiao et al., 2004; Jing et al., 2009; Bezerra et al., 2011). However, glucose concentrations in this study were too low to induce inhibition.

Due to the variations in reported grown conditions for *T. bispora*, we investigated the effect of carbon source on β-glucosidase production. Under the optimum temperature (45°C), I/S and M65 media supplemented with glucose converted 85 and 50% probe, respectively. While both I/S and M65 contain yeast extract and are considered rich media, I/S contains a number of trace elements not present in M65. The increased β-glucosidase production from I/S medium may be linked to a more robust growth environment. I/S, M65, and M9TE supplemented with MCC yielded 85,69, and 77% conversion, respectively. M9TE is a minimal medium, in which all energy must come from the hydrolysis of the provided carbon source. β-glucosidase must be produced in order for cells to breakdown MCC, resulting in high probe conversion. *T. bispora* grown on AFEX-pretreated switchgrass converted similar amounts of probe in M65 and I/S medium (80 and 66%, respectively). These data suggest carbon source has a minimal effect on β-glucosidase production, and many conditions were found to cleave the probe with efficiency.

Assay conditions for cleavage of the cellobiose were then optimized for temperature and pH. Environments that produced high β-glucosidase activity were identified from the high throughput screening and used to optimize assay conditions. Supernatants from one such condition, I/S + 10 mM glucose at 50°C for 24 h, cleaved ~85% of the NIMS probe (**Figure 2**). Cell cultures were grown to these specifications and supernatants were used to optimize the incubation protocol.

**FIGURE 4:**
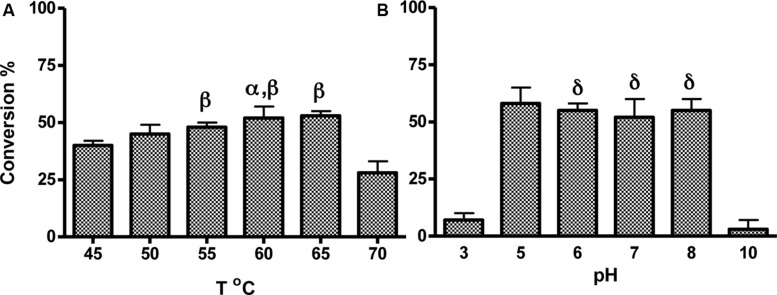
**Optimization of assay conditions with cellobiose substrate under growth conditions identified by high throughput NIMS screening.** Supernatants of cell culture grown in I/S + 10 mM glucose at 50°C for 24 h were incubated with the probe at various temperatures **(A)** or pH buffers **(B)**. Error bars represent standard deviation from the mean of triplicate technical replicates. Significance indicated by: ^α^*p*< 0.05 versus activity at 45°C, ^β^*p* > 0.05 versus activity at 50°C, ^δ^*p* > 0.05 versus activity at pH5.

To determine optimal enzyme reaction conditions, supernatant + the NIMS probe were incubated between 45 and 70°C for 1 h, then cleavage of the probe was measured. Conversion of the substrate to product increased with temperature and was optimal between 50 and 65°C (*p* > 0.05), and sharply decreased at 70°C (**Figure 4A**). In addition, assays were buffered with 10 mM salts at different pH to test enzyme stability and performance. There was no difference between assays conducted between a pH range of 5–8 (*p* > 0.05, **Figure 4B**), suggesting direct incubation of culture supernatant with probe is sufficient for optimum conversion.

In this study, the effects of temperature and carbon source on β-glucosidase production in well-defined media were explored. Different combinations of carbon sources were tested with I/S and M65 because they are two media that reportedly support growth of *T. bispora*. M9TE is minimal medium, which sustained a diverse enrichment community derived from compost when supplemented with MCC. *T. bispora* dominated these enrichments, however, the community dynamics remain unknown (Hiras, unpublished data). Therefore, changing the substrate in M9TE medium may not have sustained *T. bispora* growth and is a topic for further examination. The polysaccharide fraction in M84 is the soluble fraction derived from boiling rolled oats. We are unable to exclude the soluble polysaccharides and retain other nutrients that may emerge during the boiling process. Ultimately, enough duplicate conditions were chosen that would fit on a single 24-well plate for validation of this method. To create a more robust data set in the future, growth temperatures and carbon source variations will be expanded. Statistical analyzes will be possible after generating triplicate cultures for each condition, normalizing percent conversion to protein concentration, and including internal standards to produce qualitative data.

## CONCLUSION

A high throughput method of screening *T. bispora* for β-glucosidase production under various growth conditions was developed using NIMS coupled with acoustic deposition. This method has been shown to be suitable for analysis of crude microbial communities and resolving specific enzyme activities. The ability of *T. bispora* to grow on media containing a variety of energy sources and at specific temperatures and time were tested. This report validates NIMS as a tool to rapidly screen many conditions for production of a desired compound. The results show that nearly all cell cultures grown at 45°C had higher conversion of substrate to product than their counterparts at 50,55, and 60°C, which suggests 45°C is the optimal temperature for cellulase production after 24 h. This high throughput NIMS approach may provide an important tool in discovery and characterization of enzymes environmental microbes for industrial and biofuel applications.

## Conflict of Interest Statement

The authors declare that the research was conducted in the absence of any commercial or financial relationships that could be construed as a potential conflict of interest.
